# Exploration of workforce changes in integrated chronic care: Findings from an interactive and emergent research design

**DOI:** 10.1371/journal.pone.0187468

**Published:** 2017-12-21

**Authors:** Loraine Busetto, Katrien Luijkx, Stefano Calciolari, Laura Guadalupe González Ortiz, Hubertus Johannes Maria Vrijhoef

**Affiliations:** 1 Department of Neurology, Heidelberg University Hospital, Heidelberg, Germany; 2 Tranzo Scientific Center for Care and Welfare, Tilburg University, Tilburg, the Netherlands; 3 Faculty of Economics, University of Lugano, Lugano, Switzerland; 4 Department of Patient & Care, Maastricht University Medical Center, Maastricht, the Netherlands; 5 Department of Family Medicine and Chronic Care, Vrije Universiteit Brussel, Brussels, Belgium; 6 Panaxea BV, Amsterdam, the Netherlands; Universidade de Mogi das Cruzes, BRAZIL

## Abstract

**Introduction:**

Integrated care interventions introduced in response to the increased demand for long-term care entail profound changes to the health workforce. This exploratory study aims to provide an overview of the workforce changes implemented as part of integrated chronic care interventions.

**Methods:**

An interactive and emergent research design was used consisting of a literature review, qualitative expert questionnaires and case reports. We defined integrated care as interventions targeting at least two of the six Chronic Care Model components. Workforce changes were defined as those changes experienced by clinical and non-clinical staff responsible for public and individual health intervention.

**Results:**

Seven workforce changes were identified: (1) nurse involvement, (2) multidisciplinary staff, (3) multidisciplinary protocols/pathways, (4) provider training, (5) case manager/care coordinator, (6) team meetings, and (7) new positions. Most interventions included more than one of these workforce changes.

**Conclusion:**

The results of this study provide detailed insights into the current implementation of workforce changes in integrated care interventions and thereby pave the way for further investigations into the relative effectiveness of different workforce changes within the scope of complex interventions. Advancing knowledge in this area is essential for fostering health systems’ capacity to cope with the challenges related to the current demographic and epidemiological trends.

## Introduction

The past decades have been characterised by a growing prevalence of chronic conditions, an increasing number of older and often multi-morbid patients as well as a correspondingly rising demand for complex, long-term care [1–4]. In addition, Frenk et al. summarise the following systemic problems inherent to most current health care systems: a mismatch of competencies to patient and population needs; poor teamwork; persistent gender stratification of professional status; narrow technical focus without broader contextual understanding; episodic encounters rather than continuous care; predominant hospital orientation at the expense of primary care; quantitative and qualitative imbalances in the professional labour market; and weak leadership to improve health-system performance [5]. Unfortunately, it is becoming increasingly evident that most health systems are not sufficiently equipped to deal with these challenges. Instead, there seems to be a significant mismatch between the most prevalent health problems, i.e. increasing prevalence of (multiple) chronic diseases, and the preparation of the workforce to deal with them. As a consequence, patients with chronic conditions are often stuck in the revolving doors of multiple providers (often across care settings) who neither are adequately coordinated nor have a clear vision of where the road is–or should be–headed afterwards [6].

In response to these problems, the implementation of integrated care has become a priority in various countries. Integrated care is a means to deliver high quality long-term care to people with chronic conditions [7, 8]. It concerns complex interventions including changes to the health system, engagement of community resources, strong patient-provider relationships, care processes re-design, advanced communication infrastructures, and new approaches by health professionals to deliver care [9–11]. However, as highlighted by Stein, while there is currently much focus on the implementation and execution of integrated care strategies, there is not yet a comparable focus on those who implement and execute this strategies in their daily practice [12]. Since health professionals are involved in all aspects of care delivery, changes to the health workforce affect the implementation of integrated care profoundly. Bodenheimer and Sinsky even propose to include the health workforce in the Quadruple Aim, which is an extension of the traditional Triple Aim focusing on enhancing patient experience, improving population health, and reducing costs [13]. Given the symbiotic relationship between providers and recipients of health care, one can argue that the workforce is a prerequisite for improving patient outcomes [13, 14].

Previous studies on the health workforce have investigated health workforce planning [15, 16], assessed present and future health workforce needs [17], and predicted trends for specific sectors or groups of health professionals [18, 19]. Other studies have investigated specific types of changes for the health workforce such as skill mix or team work [20, 21]. However, none of these studies were conducted specifically on integrated care interventions for chronic diseases. This is problematic for two reasons. First, chronic care with its focus on long-term management of illness differs considerably from acute care with its focus on episodic treatment of illness. Second, when workforce changes are not implemented as single interventions, but as part of integrated care interventions, they are implemented in combination with changes targeting the other areas of integrated care delivery described above.

As a contribution to the field of integrated chronic care, the aim of the current exploratory study is to provide an overview specifically of those workforce changes that have been implemented as part of integrated care interventions for people with chronic diseases. Within the scope of a flexible and emergent research design, data were collected from a literature review, expert questionnaires and case reports. This study is part of Project INTEGRATE “Benchmarking Integrated Care in Chronic and Age-related Conditions in Europe”. Within the scope of this project, we also investigated the barriers, facilitators and outcomes of the workforce changes implemented as part of integrated care interventions. These findings are reported elsewhere [22, 23].

## Methods

Studying workforce changes as part of complex, multifaceted interventions requires the use of study designs that can capture this complexity. Because of their multifaceted nature, complex interventions must be studied from different angles, which requires the use of different data sources. However, the data collection and analysis from each of these sources might develop in unforeseen ways and their combination may therefore require changes to the research design throughout the different stages of its execution [24]). These changes can be accommodated most appropriately within the scope of a flexible and emergent design, that is, a design that evolves throughout its different stages and allows for the interaction between different strands of data at different points of time during the research [25]. This makes it possible to use preliminary findings from one data source as a basis for the subsequent data collection or as a framework for data analysis or interpretation.

This study employed an emergent and interactive research design that included a literature review, empirical research via a qualitative expert questionnaire, and secondary analysis of two case reports. The research design has been described in detail elsewhere [24].

### Definitions

In line with previous research, we defined integrated care as interventions targeting at least two of the six Chronic Care Model (CCM) components (i.e. health system, self-management support, delivery system design, decision support, clinical information system and community) [10, 11, 26]. (Health) workforce changes were defined as those changes experienced by clinical and non-clinical staff responsible for public and individual health intervention [27].

### Data collection and analysis

Dutch law does not require ethical approval for data collection among health professionals and experts. Before completing the expert questionnaire, respondents were informed about the purpose of the study and asked to provide informed consent.

#### Literature review

Between July and October 2014, a literature search was conducted using a four-step approach including a (1) systematic database search, (2) semi-systematic database search, (3) secondary analysis of a previous literature review, and (4) unsystematic hand searches. The systematic literature search was performed in PubMed/Medline, CINAHL, Science Direct, and Business Source Premiere. Three groups of search terms relating to chronic diseases, intervention type, and workforce were combined. The complete search strategy for one database is reported in Table 1 [22]. A PRISMA Checklist is provided (S1 PRISMA Checklist).

**Table 1:** Full search strategy for PubMed.

#	Category	Search terms
#1	Health condition: chronic conditions (general)	Chronic disease[Title/Abstract] OR chronic diseases[Title/Abstract] OR chronic condition[Title/Abstract] OR chronic conditions[Title/Abstract] OR comorbidity[Title/Abstract] OR co-morbidity[Title/Abstract] OR co-morbid[Title/Abstract] OR multimorbidity[Title/Abstract] OR multi-morbidity[Title/Abstract]
#2	Health condition: COPD	COPD[Title/Abstract] OR Chronic Bronchitis[Title/Abstract] OR Bronchitis[Title/Abstract] OR Emphysema[Title/Abstract]) OR Chronic Obstructive Pulmonary Disease[Title/Abstract] OR COAD[Title/Abstract] OR Chronic Obstructive Airway Disease[Title/Abstract] OR Chronic Obstructive Lung Disease[Title/Abstract] OR Chronic Airflow Obstruction[Title/Abstract] OR Chronic Airflow Obstructions[Title/Abstract]
#3	Health condition: diabetes	diabetes[Title/Abstract] OR diabetes type 2[Title/Abstract] OR diabetes mellitus[Title/Abstract] OR DMT2[Title/Abstract] OR diabetes mellitus type 2[Title/Abstract]
#4	Health condition: geriatric conditions	Geriatrics[Title/Abstract] OR Gerontology[Title/Abstract] OR Geriatric care[Title/Abstract] OR Geriatric condition[Title/Abstract] OR Geriatric syndromes[Title/Abstract] OR Geriatric syndromes[Title/Abstract] OR Frailty[Title/Abstract] OR Frail elderly[Title/Abstract] OR Geriatric assessment[Title/Abstract] OR Falls[Title/Abstract] OR Elderly[Title/Abstract] OR Older people[Title/Abstract]
#5	Integrated care	integrated care[Title/Abstract] OR disease management[Title/Abstract] OR disease state management[Title/Abstract] OR comprehensive healthcare[Title/Abstract] OR complex interventions[Title/Abstract] OR multifactoral lifestyle interventions[Title/Abstract] OR shared care[Title/Abstract] OR chronic care model[Title/Abstract] OR care transition[Title/Abstract] OR transitional care[Title/Abstract] OR intermediate care[Title/Abstract] OR case management[Title/Abstract]
#6	Workforce changes (Search 1)	Human resources[Title] OR human resource management[Title] OR skill mix[Title] OR workforce[Title] OR health workforce[Title]) OR health care workforce[Title] OR workforce change[Title] OR workforce changes[Title] OR qualifications[Title] OR staff mix[Title] OR role enhancement[Title] OR role enlargement[Title] OR role substitution[Title] OR role delegation[Title] OR staff ratio[Title] OR workforce design[Title] OR workforce redesign[Title] OR skill management[Title]) OR skill development[Title] OR skill flexibility[Title] OR up-skilling[Title]) OR side-skilling[Title] OR health personnel[Title] OR personnel staffing[Title] OR professional roles[Title] OR skill substitution[Title] OR staff skills[Title]
#7	Workforce changes (Search 2)	professional competence[Title] OR professional role[Title] OR professional skills[Title] OR professional responsibilities[Title] OR professional tasks[Title]) OR nurse-physician collaboration[Title] OR professional collaboration[Title] OR nurse practitioner[Title] OR advanced nurse practitioner[Title] OR advanced nurse specialist[Title] OR physician assistant[Title] OR Advanced care practitioner[Title] OR Care co-ordinator[Title] OR Community matron[Title] OR Link-workers[Title]
Search 1:		((#1 or #2 or #3 or #4) and #6) or (#5 and #6)
Search 2:		((#1 or #2 or #3 or #4) and #7) or (#5 and #7)
Limitations:		published after 2000

Three selection rounds based on title, abstract and full text were performed individually by three researchers (LB, SC, LG) and then discussed together until consensus was reached. Articles were included when they focused on the health workforce, integrated care and chronic diseases/care and were published after 2000, given the increased focus on integrated care over the past 15 years [8]. Articles were excluded when they were published in a language other than English, Dutch, German, Italian or Spanish, were conducted in a developing country or concerned non-empirical research. Systematic reviews and meta-analyses were excluded because experience with a previous review showed that the majority of reviews and meta-analyses based their findings on interventions that did not (all) fit our definition of integrated care.

The initial, limited output of the systematic database search was reported to the scientific committee of Project INTEGRATE, consisting of senior researchers from eight different European countries, who provided us with another set of health workforce related search terms. These search terms were combined in the previously used search string with the integrated care and chronic care related search terms and the search was repeated in PubMed, CINAHL and ScienceDirect. We did not conduct an additional search in Business Source Premier because of the very low number of relevant articles resulting from the initial search. The results of the search were assessed in a semi-systematic way based on the in- and exclusion criteria described earlier. By semi-systematic we mean that one researcher (instead of two independent researchers) performed the title and abstract selection, while suggestions for inclusion were discussed by three researchers (LB, SC, LG).

As a third step, we re-assessed a previous literature review on integrated care for type 2 diabetes that followed a similar approach to the current review [28]. All articles included in the previous review focused on integrated care and a chronic disease. Articles were checked for a focus on health workforce changes and were included if applicable. Finally, all researchers conducted unsystematic hand searches of the reference lists of articles obtained from the previous search steps and via Google. Articles suggested for inclusion were assessed and discussed by three researchers (LB, SC, LG) until consensus was reached. For example, it was discussed whether the article in question described a “real” intervention that had been implemented or rather a theoretical article concerning the literature, simulations and forecasts [29, 30]. Another point of discussion was whether interventions where implemented in the chronic or acute care setting [16].

The data extraction was performed between September 2014 and October 2014 independently by two researchers and then compared in pairs (LB and SC, LB and LG, SC and LG). A list of common workforce changes was compiled by one researcher (LB) and checked independently by two researchers (SC, LG). The list was discussed and adapted until all researchers agreed that all workforce changes from the included studies were covered and there were no redundancies in the list. It was not possible to provide comprehensive definitions of the workforce changes based on the limited information available in the studies. Instead, we provided a succinct description for each type of workforce change in order to ensure a uniform understanding and application of the respective concepts.

#### Expert questionnaires

Between January and April 2015, a qualitative exploratory questionnaire was sent to experts on integrated care, chronic care, and health human resource management. Respondents were recruited using the snowball method, including experts with academic or policy backgrounds as well as field experts (i.e. health professionals or managers of organisations involved in the provision of integrated care). Experts had to be proficient in written language in one of the languages in which the questionnaire was available (i.e. English, Dutch, Italian and Spanish). Experts were asked to describe an integrated care intervention and the workforce changes included in this intervention. These descriptions were mapped by the authors to the 11 workforce changes coded in the literature review. In a separate question, the experts were requested to indicate which of the workforce changes from the literature review they recognised from their own experience [22]. In order to yield a satisfactory response rate, two rounds of email reminders were sent to non-responders. Due to the international scope of the questionnaire, we did not expect that conducting the questionnaire by mail or telephone would contribute to increasing the response rate as respondents may have been unwilling or unable to pay the postage or speak a non-native language over the phone.

The English questionnaire was translated to three target languages (Dutch, Italian, and Spanish) according to the languages in which at least one of the authors of this article is a native speaker. Based on a feasible adaption of recommendations provided in the relevant scientific literature [31–34], we opted for the following pragmatic multi-step approach: (1) original English questionnaire checked by a native speaker of English; (2) forward translations by native speakers of the target language; (3) back translations to English by a researcher proficient in English; and (4) discussion of English versions (original and back translation). The translation of “workforce changes” was a problematic issue because no precise or unambiguous translation could be agreed upon in Dutch, Spanish, and Italian. Eventually, the researchers agreed on using the best available approximate translation and adding several examples of workforce changes to further clarify the concept. These examples were the same in all translations.

The coding was performed by four researchers (LB, KL, SC, LG). The list obtained from the literature review was used as initial coding list for the coding of the workforce changes. The coding list was expanded and adapted when necessary after discussion among the coders.

#### Case reports

Two detailed reports of case studies conducted from September 2012 to March 2014 as part of Project INTEGRATE, were available for secondary analysis. The first case report described the implementation of integrated care for geriatric conditions at a German geriatric hospital [35]. This hospital consists of five wards, each organised in independent multidisciplinary teams consisting of doctors, physiotherapists, occupational therapists, nurses and neuropsychologists [35]. The second case report concerned the implementation of integrated care for type 2 diabetes mellitus by two Dutch care groups. Care groups are legal entities that establish contracts with health insurers and health professionals in order to coordinate the so-called ‘care chain’ of chronic care from diagnosis to after care [36]. A scientific paper based on the detailed report was published elsewhere [37].

The workforce changes described in the case reports were mapped by one researcher (LB) to the coding list identified from the literature review described above. The data extraction and the mapping of workforce changes were sent to the authors of the German case report for feedback. Changes and comments were taken up in the analysis of the case reports. This check was not performed for the Dutch case since the authors of the Dutch case are also the authors of the current study.

## Results

### General information

#### Literature review

**Fig 1:**
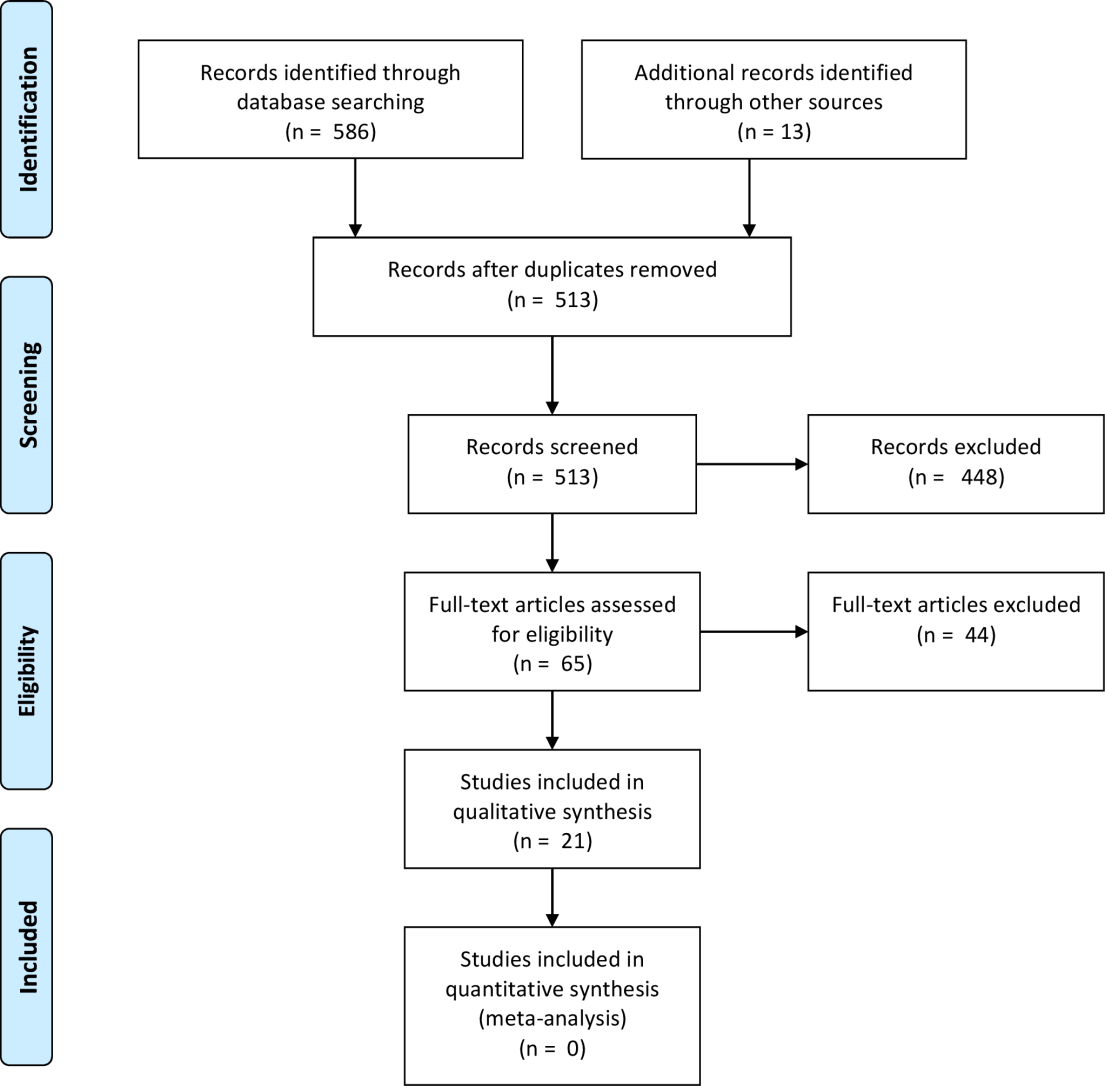
Flowchart of the literature review selection process.

Fig 1 depicts the selection process of the four phases of the literature review. The final selection consisted of 21 studies.

The study characteristics of the studies included in the literature review are presented in Table 2. The interventions described in the studies were implemented in seven countries, including the United States (N = 10), the Netherlands (N = 4), the United Kingdom (N = 2), Canada (N = 2), Belgium (N = 1), Austria (N = 1), and Germany (N = 1). Interventions were implemented for patients with diabetes (N = 16), patients with any type of chronic disease (N = 3), older patients with dementia and/or depression (N = 1), and patients with rheumatoid arthritis (N = 1).

**Table 2:** Study characteristics of studies included in the literature review.

Ref	Title	Year	Country	Condition	Objective
[38]	Implementation and methodology of a multidisciplinary disease-state-management program for comprehensive diabetes care	2011	United States	Diabetes	To implement and evaluate a multidisciplinary disease-state-management program for comprehensive diabetes care
[39]	The impact of a staged management approach to diabetes foot care in the Louisiana public hospital system	2003	United States	Diabetes	To evaluate the effect of a staged-management approach on foot-related hospitalizations and lower extremity amputations
[40]	Interdisciplinary diabetes care teams operating on the interface between primary and specialty care are associated with improved outcomes of care: findings from the Leuven Diabetes Project	2009	Belgium	Diabetes	To create the basis for the development of a national diabetes care program
[41]	Best practices in innovative type 2 diabetes program management: a case study. Journal of managed care pharmacy	2005	United States	Diabetes	To illustrate a successful type 2 diabetes management program
[42]	An evaluation of a Diabetes Specialist Nurse prescriber on the system of delivering medicines to patients with diabetes	2008	United Kingdom	Diabetes	To evaluate the impact of a Diabetes Specialist Nurse prescriber on insulin and oral hypoglycaemic agent medication errors and length of stay
[43]	A patient-centric, provider-assisted diabetes telehealth self-management intervention for urban minorities	2011	United States	Diabetes	To describe the design, implementation, and outcomes of a pilot self-management intervention targeting urban African Americans with type 2 diabetes
[44]	New Workforce Development in Dementia Care: Screening for “Caring”: Preliminary Data	2014	United States	Dementia and/ or depression	To describe the applicant (to a care coordinator assistant position) screening and hiring process and outcomes
[45]	An evaluation of a specialist nurse prescriber on diabetes in-patient service delivery	2007	United Kingdom	Diabetes	To evaluate the impact of a diabetes specialist nurse prescriber on insulin and medication errors, length of stay and the ability of patients to self-manage whilst in hospital
[46]	IT-supported skill-mix change and standardisation in integrated eyecare: lessons from two screening projects in The Netherlands	2007	The Netherlands	Diabetes	To explore the possibilities of creating an optimal fit between skill-mix change and IT through standardization
[47]	Nurse-led shared care diabetes projects: lessons from the nurses' viewpoint	2003	Netherlands	Diabetes	To explore nurses’ experiences with shared care
[48]	Effectiveness of the Austrian disease management programme "Therapie Aktiv" for type 2 diabetes regarding the improvement of metabolic control, risk profile and guideline adherence: 2 years of follow up	2012	Austria	Diabetes	To assess the prolonged impact of a disease management programme regarding HbA1c reduction and process quality
[49]	Effect of nurse practitioner and pharmacist counseling on inappropriate medication use in family practice	2012	Canada	Any type of chronic condition	To explore the effects of a multidisciplinary care intervention on medication use for geriatric patients
[50]	Nurse case management improves blood pressure, emotional distress and diabetes complication screening	2006	United States	Diabetes	To measure the impact of a patient-oriented structured approach to care coordination and patient education and counseling
[51]	Nurse Practitioner Co-Management for Patients in an Academic Geriatric Practice	2013	United States	Any type of chronic condition	To evaluate a quality improvement program that compared usual primary care by academic geriatricians with care co-managed by a Nurse Practitioner
[52]	Training community health workers as diabetes educators for urban African Americans: value added using participatory methods. Progress in community health partnerships: research, education, and action	2007	United States	Diabetes	To describe the community health workers recruitment, training, and evaluation procedures utilized in Project Sugar 2
[53]	The challenge of promoting integration: conceptualization, implementation, and assessment of a pilot care delivery model for patients with type 2 diabetes	2004	Canada	Diabetes	To describe the development and implementation process of a new delivery system and to describe the preliminary findings from the evaluation
[54]	Development of a diabetes care management curriculum in a family practice residency program	2004	United States	Diabetes	To describe the implementation of a diabetes disease management team, to discuss the methods used to overcome the described barriers, and to provide a qualitative assessment of learner’s evaluation of the process
[55]	Readmissions of patients with diabetes mellitus and foot ulcers after infra-popliteal bypass surgery—attacking the problem by an integrated case management model	2013	Germany	Diabetes	To study the effects of an integrated case management system for patients with Diabetic Foot Syndrome on readmission rates, length of stay, and hospital costs
[56]	Effects of a nurse practitioner on a multidisciplinary consultation team	2009	Netherlands	Rheumatoid arthritis	To evaluate the impact of a nurse practitioner on office hours capacity, patient satisfaction, quality of life and costs
[57]	The nurse specialist as main care-provider for patients with type 2 diabetes in a primary care setting: effects on patient outcomes	2002	Netherlands	Diabetes	To provide evidence about a shared care model for patients with type 2 diabetes when the diabetes nurse was the main care-provider
[58]	Nurse practitioner-led multidisciplinary teams to improve chronic illness care: The unique strengths of nurse practitioners applied to shared medical appointments/group visits	2008	United States	Any type of chronic condition	To describe the roles of nurse practitioners in shared medical appointments/group visits

#### Expert questionnaires

The questionnaire was sent to 91 scholars and professionals. Overall, 25 recipients completed and returned the questionnaire, resulting in an overall response rate of 28%, which seems to be in line with average response rates for online surveys [59–61]. The interventions described by the respondents were implemented in 12 different countries, including Belgium (N = 8), Spain (N = 5), Estonia (N = 2), Italy (N = 2), the Netherlands (N = 2), the United Kingdom (N = 2), Australia (N = 1), Czech Republic (N = 1), Germany (N = 1), Greece (N = 1), Norway (N = 1), and Switzerland (N = 1). Most often, interventions were implemented for patients with any type of chronic/long-term illnesses (N = 5), patients with diabetes (N = 5), COPD patients (N = 4), people with cardiovascular disease (N = 2), and all patients (N = 2).

### Workforce changes

#### Literature review

The following eleven workforce changes were identified from the included articles:

- *Nurse-led care/nurse as main care provider*: a nurse is the main care provider for the patient and/or the team is led by the nurse;
- *Multidisciplinary protocols/pathways*: care is delivered according to protocols or pathways that involve tasks for health professionals from different disciplines or with different medical specialties;
- *Multidisciplinary staff*: care is delivered by a team that includes health professionals from different disciplines or with different medical specialties;
- *Nurse involvement*: a nurse is involved in the delivery of care;
- *Pharmacist involvement*: a pharmacist is involved in the delivery of care;
- *Team meetings*: a care team that works around a patient or group of patients meets on a regular basis to discuss the patients’ treatment;
- *Case manager/care coordinator*: a case manager or care coordinator or someone assuming a similar role is involved in the delivery of care;
- *Provider training*: on-the-job training or educational seminars or materials are provided to health professionals;
- *New position*: a new position, role or function is created specifically to deliver integrated chronic care;
- *Task re-distribution*: the tasks of health professionals involved in the delivery of care are re-distributed;
- *Shared medical appointments*: consultations are delivered by different health professionals during the same appointment.

Table 3 shows an overview of the workforce changes described in the respective studies. A mean number of 2.81 workforce changes was described per study (M = 2.81; SD = 1.17). In two studies, only one workforce change was included. Nurse involvement is the workforce change described most frequently in the literature (N = 19; 91%), followed by multidisciplinary staff (N = 11; 52%). They are also often implemented in combination (N = 10; 48%).

**Table 3:** Overview of workforce changes per included study and number and percentage of studies mentioning the respective changes.

Workforce changes	Studies																						
	[39]	[40]	[41]	[42]	[43]	[44]	[45]	[46]	[47]	[48]	[49]	[50]	[51]	[52]	[53]	[54]	[55]	[56]	[57]	[58]	[59]	n	%
Nurse-led care/nurse as main care provider	x									x						x				x	x	**5**	**24**
Multidisciplinary protocols/pathways		x							x	x	x				x				x			**6**	**29**
Multidisciplinary staff		x	x	x					x	x	x	x				x	x		x		x	**11**	**52**
Nurse involvement	x	x	x	x	x	x		x	x	x		x	x	x	x	x	x	x	x	x	x	**19**	**91**
Pharmacist involvement				x								x										**2**	**10**
Team meetings				x																		**1**	**5**
Case managers/care coordinators							x			x					x	x		x	x			**6**	**29**
Provider training					x			x														**2**	**10**
New position							x		x													**2**	**10**
Task re-distribution							x		x					x						x		**4**	**19**
Shared medical appointments																					x	**1**	**5**

Notes: X indicates that the workforce change was mentioned in the respective study. Empty cells indicate that the workforce change was not mentioned in the respective study. Abbreviations: n = number of studies, % = percentage of total number of studies.

Both times the introduction of a new position was described, task-redistribution was described as well. Case managers/care coordinators were only once described in combination with the introduction of a new position, and also only once in combination with task-redistribution. This suggests that the other four times case managers/care coordinators were described, other workforce changes were insufficiently described, because, logically, either the introduction of a new position or the re-distribution of tasks must have taken place along with the introduction of a care manager/care coordinator role, but neither was described explicitly in those studies.

Nurse-led care/nurse as main care provider was always described together with nurse involvement, but not the other way around. This suggests that not all nurses involved had a leading role (or that this was not reported). Team meetings were mentioned only once, even though multidisciplinary staff and protocols were mentioned much more frequently (11 and six times, respectively). Multidisciplinary staff and protocols/pathways were described together five times, but no study mentioned these two changes and team meetings together. This could mean that those interventions indeed involved professionals from different professional backgrounds who worked together according to protocols and pathways outlining their interactions or tasks, but who do not hold regular team meetings together. Alternatively, it could results, again, from under-reporting. In general, while there is some room to check for logical consistency, we cannot be entirely sure whether (for example) an intervention including nurse involvement did really not include task redistribution as well, or whether we are faced with an under-reporting of elements included in the interventions. To gain these kinds of insights, more detailed quantitative as well as qualitative methods would have to be employed in future.

#### Expert questionnaires

Table 4 shows which workforce changes were described by the respondents (the numbers in the second row indicate the 25 experts). The experts described interventions with a mean number of 1.72 workforce changes per intervention (M = 1.72; SD = 0.84). This is lower than the number of workforce changes per integrated care interventions described in the studies.

**Table 4:** Overview of the workforce changes described by the questionnaire respondents and number and percentage of respondents mentioning the respective changes.

Workforce changes	Experts																										
	1	2	3	4	5	6	7	8	9	10	11	12	13	14	15	16	17	18	19	20	21	22	23	24	25	n	%
Nurse-led care/nurse as main care provider	x																				x					**2**	**8**
Multidisciplinary protocols/pathways								x		x		x			x									x		**5**	**20**
Multidisciplinary staff		x	x					x	x							x			x		x	x	x	x	x	**11**	**44**
Nurse involvement	x				x		x											x			x					**5**	**20**
Pharmacist involvement																										**0**	**0**
Team meetings																									x	**1**	**4**
Case managers/care coordinators	x															x				x	x					**4**	**16**
Provider training			x	x		x					x		x	x			x							x		**8**	**32**
New position		x			x		x							x						x						**5**	**20**
Task re-distribution														x								x				**2**	**8**
Shared medical appointments																										**0**	**0**

Notes: X indicates that the workforce change was mentioned. Empty cells indicate that the workforce change was not mentioned by the respective expert. Abbreviations: n = number of respondents, % = percentage of respondents.

Multidisciplinary staff (N = 11; 44%), provider training (N = 8; 32%), multidisciplinary protocols/pathways (N = 5; 20%), and creation of a new position (N = 5; 20%) were described most often by the respondents. In addition to the workforce changes presented in Table 4, seven respondents described other approaches, tools and guidelines to support the delivery of care that did not fit a common category.

Again, nurse-led care/nurse as main care provider was always described together with nurse involvement, but not the other way around. And again, team meetings were mentioned only once, even though multidisciplinary staff and protocols were mentioned much more frequently (11 and five times, respectively). Multidisciplinary staff and protocols/pathways were described together less frequently than in the studies (only twice), and again, no study mentioned these two changes and team meetings together.

Of the four times case managers/care coordinators were described, they were described only once in combination with the creation of a new position and never in combination with task-redistribution, again suggesting an under-reporting of workforce changes. Provider education was described more often by the experts (32%) than in the studies (10%). Shared medical appointments were described only once in the studies, but not at all by the experts.

As previously mentioned, the respondents were also presented with the 11 workforce changes identified in the literature review and asked which of these changes they recognised from their own expertise or experience. Table 5 shows which workforce changes were confirmed by the experts (the numbers in the second row indicate the 25 experts). The experts confirmed a mean number of 6.36 workforce changes per expert (M = 6.36; SD = 3.17). The average number of workforce changes confirmed per expert is much higher than those described per study or expert because in the former case the experts were asked about all workforce changes they were familiar with *in general*, while in the latter case workforce changes were described *per intervention*.

**Table 5:** Overview of the workforce changes confirmed by the questionnaire respondents and number and percentage of respondents confirming the respective changes.

Workforce changes	Experts																										
	1	2	3	4	5	6	7	8	9	10	11	12	13	14	15	16	17	18	19	20	21	22	23	24	25	n	%
Nurse-led care/nurse as main care provider	x	x			x	x	x	x						x			x	x			x				x	**11**	**44**
Multidisciplinary protocols/pathways	x	x	x		x		x	x	x	x		x	x	x	x		x	x	x	x	x	x	x	x	x	**21**	**84**
Multidisciplinary staff	x	x	x		x		x	x	x				x	x	x		x	x	x	x	x	x	x	x	x	**19**	**76**
Nurse involvement	x	x		x	x	x	x	x	x			x		x	x		x	x	x	x	x	x		x	x	**19**	**76**
Pharmacist involvement	x	x			x		x	x	x						x					x	x		x			**10**	**40**
Team meetings	x	x	x	x	x	x	x	x	x			x	x	x			x	x	x	x	x	x	x		x	**20**	**80**
Case managers/care coordinators	x	x					x	x	x					x	x		x	x	x	x	x	x	x			**14**	**56**
Provider training	x		x	x		x	x	x	x					x	x		x		x	x		x	x		x	**15**	**60**
New position	x	x					x	x			x			x					x	X	x				x	**10**	**40**
Task re-distribution	x						x	x	x				x	x	x			x			x	x				**10**	**40**
Shared medical appointments	x				x		x	x			x		x	x						x	x		x			**10**	**40**

Notes: X indicates that the workforce change was confirmed. Empty cells indicate that the workforce change was not confirmed by the respective expert. Abbreviations: n = number of respondents, % = percentage of respondents.

Multidisciplinary protocols/pathways were confirmed by most experts (N = 21; 84%), followed by team meetings (N = 20; 80%), multidisciplinary staff (N = 19; 76%), and nurse involvement (N = 19; 76%). Again, nurse involvement was confirmed more often than nurse-led care/nurse as main care provider. Shared medical appointments and team meetings which were described in the studies and by the experts only zero or one time, were now confirmed ten and 20 times, respectively. There were three experts who confirmed all 11 workforce changes. Two experts recognized all workforce changes except for provider education and pharmacist involvement, respectively. Respondent 16 did not confirm any of the workforce changes, despite having described two workforce changes in response to the first question (see Table 4).

#### Case reports

A secondary analysis was performed for the German and Dutch case reports described above. The workforce changes in the integrated care interventions described in the reports case were mapped to the list of 11 workforce changes from the literature review. Table 6 shows which case studies incorporated which of the workforce changes identified in the literature review.

**Table 6:** Overview of workforce changes per case report and number of case reports mentioning the respective changes.

Workforce changes	Case reports		
	Germany	Netherlands	n
Nurse-led care/main care provider		x	**1**
Multidisciplinary protocols/pathways		x	**1**
Multidisciplinary staff	x	x	**2**
Nurse involvement	x	x	**2**
Pharmacist Involvement			**0**
Team meetings	x		**1**
Case managers/care coordinators		x	**1**
Provider training		x	**1**
New position		x	**1**
Task re-distribution		x	**1**
Shared medical appointments			**0**

Notes: X indicates that the workforce change was present in the respective case report. Empty cells indicate that the workforce change was not present in the respective report. Abbreviations: n = number of case reports.

Multidisciplinary staff and nurse involvement were present at both case sites (N = 2). The Dutch case report mentioned nurse-led care/nurse as main care provider, multidisciplinary protocols/pathways, case managers/care coordinators, provider training, new position and task-redistribution (N = 1). The German case report mentioned team meetings (N = 1). Shared medical appointments were present in neither of the cases (N = 0). Pharmacist involvement was planned in the Netherlands but had not yet been implemented at the time the case study was conducted.

#### Synthesis

We compared the workforce changes that were among those mentioned by most studies, experts or cases. For the literature review and expert questionnaires, this was evidenced by their belonging to the three highest percentages per data source. Given the low number of case reports and consequent distribution of percentages, we only included those workforce changes that were present in both case reports. Table 7 presents an overview of the workforce changes that were among those mentioned by most studies, experts or cases.

**Table 7:** Overview of the workforce changes among the highest three percentages in the literature review or expert questionnaires, or present in both case reports.

Workforce changes	Literature Review	Case Reports	Expert Questionnaire Description	Expert Questionnaire Confirmation
Nurse involvement	**91%**	**100%**	**20%**	**76%**
Multidisciplinary staff	**52%**	**100%**	**44%**	**76%**
Multidisciplinary protocols/pathways	**29%**	50%	**20%**	**84%**
Provider training	10%	50%	**32%**	60%
Case managers/care coordinators	**29%**	50%	16%	56%
Team meetings	5%	50%	4%	**80%**
New position	10%	50%	**20%**	40%

Notes: Percentages in bold print indicate that the respective workforce change was among the highest three percentages in the literature review or expert questionnaires, or present in both case reports. Percentages in normal print indicate that the respective workforce change was not present in both case reports or among the three highest percentages in one of the other data sources.

Nurse involvement and multidisciplinary staff were mentioned in both case reports and among the highest percentages in the literature review and expert questionnaires. Multidisciplinary protocols/pathways were among the highest three percentages in the literature review and expert questionnaire (both described and confirmed). Provider training, case managers/coordinators, team meetings and new position were among the three highest percentages in either the literature review or the expert questionnaires.

After combining the results from the three different data sources, we arrive at a list of seven workforce changes that were among those mentioned by most studies, experts or cases. These can be broadly categorised according to whether they concern staff mix or workflow aspects, as organisations must not only identify the best staff mix and assemble a group of providers accordingly, they must also determine the workflow of how this group of providers cooperates and delivers care in practice [62].

Staff mix:

- Case manager/care coordinator;
- Multidisciplinary staff;
- New position;
- Nurse involvement;

Workflow:

- Multidisciplinary protocols/pathways;
- Provider training;
- Team meetings.

## Discussion

The aim of this study was to provide an overview of the workforce changes implemented as part of integrated care interventions for people with chronic diseases. To this purpose, three methods of data collection were combined, namely a literature review, expert questionnaires and case reports.

This study identified seven workforce changes that were implemented as part of integrated care interventions for people with chronic diseases. These included (1) *nurse involvement* in the delivery of care; (2) *multidisciplinary staff* including health professionals from different disciplines; (3) *multidisciplinary protocols/pathways* involving tasks for health professionals from different disciplines; (4) *provider training* such as on-the-job training or educational seminars or materials for health professionals; (5) involvement of a *case manager/care coordinator* role in the delivery of care; (6) regular *team meetings* to discuss a patient’s treatment; and (7) the creation of a *new position*, role or function specifically to deliver integrated chronic care. In practice, these workforce changes are often related to one another and implemented in combination.

Two related changes, namely team care and role change, were also identified by a recent literature review to construct a typology of workforce models used by primary care practices [63]. The authors concluded that primary care, where integrated care often takes place, would have to be team care and that workforce innovation required new human resources. The creation of new roles was also identified by a recent study on effective workforce practice in integrated health care as well as a scoping study by the British National Health Service (NHS) on best practices for integrated care for older adults [64, 65]. A global shift towards team care was also found by an international expert consultation involving experts from the United States, Canada, Australia, England, Germany and the Netherlands [66]. The same study found nurses to be the main non-physician health professionals working along doctors in primary care. Provider education was described as a facilitator in a systematic review that also reported that inter-organisational and inter-sectoral multidisciplinary provider education was necessary to underpin integrated clinical care [67]. Moreover, a recent WHO report stressed the importance of initial as well as ongoing multidisciplinary education in strengthening the future integrated care workforce [14]. This also shows the connection between the seven workforce changes which are seldom implemented in isolation, a finding that was also confirmed by other studies [68, 65]. Finally, the involvement of case managers or case coordinators was also found by the NHS scoping review mentioned earlier [65].

Some workforce changes are implemented together so often that it becomes difficult to disentangle them. This might explain why team meetings are *confirmed* by 80% of the experts but only *described* by 4%. The latter percentage may not necessarily mean that only 4% of the interventions described by the experts included team meetings, but most likely that the experts did not explicitly mention this workforce change because the concept is so similar that one might assume that it is already implicitly covered by, for example, multidisciplinary staff. Based on this line of reasoning we might assume that the average number of workforce changes per intervention described in studies and by experts is probably an underestimation of the real number, with the most obvious ones not having been mentioned explicitly. In the case of the descriptions by the experts this underreporting was possibly even further exacerbated by having to write down everything in detail, sometimes not even in one’s mother tongue. However, based on these findings, researchers might conclude that team meetings are only rarely implemented in practice even though the very opposite could be the case. To prevent this, it would be necessary to have a common terminology with clear distinctions between and descriptions of its categories. Future research should focus on increased standardisation in the terminology regarding workforce changes to generate further knowledge in this research field and to make useful recommendations for the practice setting.

The current lack of a common terminology related to workforce changes was the reason for one of the main limitations of this study. In the case of the literature review, we were surprised by the extremely low number of studies found via the systematic search (N = 2), despite our use of a rather extensive list of search terms. It is not clear whether this paucity of research found reflects a *real* paucity of research on the topic or whether the research that does exist is too difficult to find. The latter scenario might be due to a limitation that is specific to research on integrated care: there is no common definition or understanding of what constitutes integrated care [69, 70]. We identified an intervention as integrated care when it included at least two CCM components. However, this approach might have led us to exclude interventions which could have constituted integrated care but could not be categorised as such based on the limited information provided. For example, self-management interventions might be internet- or mobile device based (and therefore also target the clinical information system component) or be initiated only after the providers themselves were trained on the topic (and thereby also target the decision support component). But if this is not explicitly mentioned, it is not possible to identify the intervention as integrated care. However, currently, this approach seems to be the best available option, as evidenced by its use in the literature [28, 71–73] and confirmation by the Scientific Committee of Project INTEGRATE. On the upside, the approach ensured that no false-positives were included in our study. Moreover, we were able to use the approach for all three data sources, which ensured consistency.

Even though the inclusion of additional, mostly nurse-related, search terms led to the retrieval of several additional articles, most articles were found via the previous diabetes review which was conducted without specific focus on workforce related search terms. It was therefore difficult to gauge to what extent the list of workforce changes we found in the literature was coincidental, representative or complete. It should also be mentioned that we did not conduct a quality assessment of the methodology of the studies included in the review. This was mainly due to the difficulty in comparatively assessing the methodological quality of studies whose research designs and scopes differed considerably one from another. However, given the exploratory nature of this research, it seemed more important to find as many relevant studies as we could, rather than applying a strict but probably inconsistent quality tool. To remedy the above shortcomings, we conducted more research on the original list of workforce changes via the expert questionnaire and case studies. This showed that the list from the literature review was a good starting point for further research but indeed needed further exploration. Further quantitative investigation of the topic could provide more insights into the frequency with which the workforce changes are implemented and which of them are associated with better health outcomes.

The current study is also characterised by several strengths, the first being its interactive and emergent design, which allowed us to combine different data strands at different points of time in the data collection and analysis. This made it possible to build upon insights gained from earlier data strands and further explore concepts that became apparent during early data collection and analysis phases. The use of multiple methods of data collection mitigates their respective limitations while reinforcing their strengths [74]. Another strength of the study lies in its international scope. Our dataset includes countries that are not often represented in studies on integrated care such as Estonia, Czech Republic, Greece and Norway. Moreover, only about a third of the data were collected from the United States, the United Kingdom, Canada and Australia, which are countries that are typically over-represented in these types of studies [64]. Finally, over the past 1.5 years, the methods and results of this study were regularly fed back to and commented on by the scientific committee and advisory board members of Project INTEGRATE, which improved the quality of the study and ensured that its focus stayed in line with the current needs of the academic and practice fields of integrated chronic care.

A source of tension throughout the whole study was the dilemma between on the one hand wanting to disentangle a complex intervention such as integrated care into its components, but on the other hand to still consider the workforce changes as only a part of a bigger intervention. The former step is necessary to be better able to analyse, identify and categorise integrated care and its components, but the latter step is also essential because integrated care as a whole is assumed to be more than the sum of its disentangled parts. In the current study, our focus was mainly on the first part, that is, to zoom into one component and gain detailed insights. In this aim we were successful, but we had to discover that this happened to a certain extent at the expense of losing sight of the bigger picture. Zooming back out is not a straightforward option because one lacks the necessary information to connect the dots between those aspects of integrated care that were studied and those that were not. An issue that further complicates this conundrum is the fact that even though we disentangled integrated care and focussed on the health workforce aspects only, it turned out that health workforce interventions are also complex in their own right, as evidenced by the existence of at least seven different workforce changes, of which on average two are implemented per integrated care intervention. This makes their implementation even more complicated, as well as the evaluation thereof.

Promising approaches to address these challenges include complex typologies that are explicit about the constituent components of the overarching intervention [75] or comprehensive analytical approaches that investigate the impact of an overall intervention as well as its components [37]. This appears to be one of the most important areas for future research, namely to address the tension of needing to zoom into one specific aspect in order to know what exactly one studies but at the same time to zoom out in order to not lose sight of the bigger picture. Finding a solution to these challenges is a prerequisite for the investigation of the effectiveness of integrated care as a whole as well as its single parts in relation to each other and in relation to the whole intervention.

By identifying workforce changes with regard to how to build a multi-professional teams (i.e. staff mix) and how these teams can work together in practice (i.e. improving workflow), this paper underscores the necessity of focusing on an even earlier stage of implementing workforce changes, namely education and training. Only the development of competencies for delivering integrated care can ensure that the necessary health professions enter the workforce and are prepared to deliver care effectively together. It will also help to lessen resistance, resignation or disregard [5] which health professionals may exhibit in response to these profound changes to the way they were taught to deliver high quality care. According to Langins and Borgermans, key competencies for providing coordinated and integrated health services include patient advocacy, effective communication, team work, people-centred care and continuous learning–in addition to competencies held by patients [14]. Changing curricula to actively teach those competencies for integrated care is especially important as in most countries training still relies on models that emphasize diagnosis and treatment of acute diseases [76]. Or as Frenk et al. put it: fragmented, outdated, and static curricula (…) produce ill-equipped graduates [5]. Even though teaching competencies, building teams and working together in practice seem to be sequential phases of a linear process; their development is in fact circular and iterative. Competencies can and should be learned and taught both in schools and in practice settings; the staff mix of a team is necessarily restricted and defined by which professions are entering the workforce and which skills they acquire during their professional carriers; and the developed and executed models of cooperation determine which competencies are deemed essential and might be necessary to teach in the practice setting. It is therefore necessary to approach workforce holistically and consistently. Our study provides both a foundation as well as a constructive call to action to do so.

## Conclusion

This study provided an overview of the workforce changes implemented as part of integrated care interventions for people with chronic diseases. Generally, seven workforce changes have been implemented, namely nurse involvement, multidisciplinary staff, multidisciplinary protocols/pathways, provider training, case manager/care coordinator, team meetings and the creation of new positions. On average, integrated care interventions included two workforce changes, and possibly even more when taking into account underreporting. Certain combinations of workforce changes are implemented together more often than others, such as multidisciplinary staff and multidisciplinary protocols. The results of this study provide a solid basis for further investigations of the relative effectiveness of different workforce changes within the scope of complex interventions. Overall, it was found that research on workforce changes is difficult to access and not yet described in the literature in a systematic way. This seems to be in stark contrast with the relevance attributed to this field by the international research and practice community of chronic and integrated care. The development of a uniform and well-described terminology related to workforce changes in integrated care interventions is therefore recommended. Advancing knowledge in the area of workforce changes in integrated care interventions would help decision makers to design more appropriate integrated care interventions and foster nations’ health systems’ capacity to cope with the challenges associated with the current demographic and epidemiological trends.

## Supporting information

S1 PRISMA Checklist (DOC)
